# Perceptions of Changes in the Operating Environment and Their Impact on Occupational Health Care Practices: A Qualitative Interview Study

**DOI:** 10.1177/11786329261447088

**Published:** 2026-05-05

**Authors:** Sari Nissinen, Mari-Anne Wallius, Erja Sormunen

**Affiliations:** 1 Finnish Institute of Occupational Health, Helsinki, Finland

**Keywords:** occupational health services, health professionals, remote services, remote work, multidisciplinary collaboration, focus group interviews

## Abstract

**Background:**

Occupational health services in Finland have undergone changes due to digitalization, remote work, and evolving professional roles, all of which challenge traditional service models and require adaptation from occupational health professionals. In addition, increasing mental health demands have created new expectations of occupational health services. However, the implications of these changes for occupational health care practices are not sufficiently understood.

**Objectives:**

To explore how occupational health professionals perceive the impact of changes in the operating environment, such as digitalization, remote work, increasing mental health demands, and evolving professional roles, on occupational health care practices.

**Design:**

A qualitative interview study using inductive content analysis.

**Methods:**

Data were collected through 8 focus group interviews with 34 occupational health professionals from 8 service providers. The interviews were analyzed using inductive content analysis to identify themes related to changes in services.

**Results:**

Six main themes emerged: strengthened client orientation in occupational health service delivery, adaptation of health care practices to multilocation client organizations, technology-driven development of occupational health practices, diversified remote occupational health practices, strengthened mental health support structures, and transformed role of the occupational health physiotherapist in service delivery. Remote services have become routine, improving the accessibility of occupational health services. Multidisciplinary collaboration has shifted to virtual platforms, enabling broader participation. Mental health services have expanded, and occupational health psychologists now play a more active role in both individual and organizational support. The redefined role of occupational health physiotherapists has changed their scope of practice and improved the flexibility of and access to services.

**Conclusion:**

Occupational health professionals are responding to environmental changes by reshaping their service practices. The results highlight a shift toward more client-centered, flexible, and collaborative service models, supported by digital tools and evolving professional roles. These adaptations contribute to the development of sustainable and responsive occupational health services.

## Introduction

In Finland, occupational health (OH) care is part of the national health care system and focuses specifically on promoting employees’ work ability and preventing occupational diseases. Over 2 million employees in Finland (90% of the workforce) are covered by OH services (OHS).^1^ These services are provided by OH professionals, such as physicians, nurses, and physiotherapists, who refer clients to appropriate experts when needed, for example, to OH psychologists.^2^ OH activities are typically divided into workplace-focused and individual-focused services. Workplace-focused services include workplace surveys that assess how working conditions affect employees’ health and work ability, whereas individual-focused services consist of health examinations, medical care, and work ability negotiations (ie, consultations between the employee, employer, and OHS). Multidisciplinary collaboration is a central element of effective and client-oriented OHS provision.^3,4^

In recent years, the OH operating environment in Finland has undergone significant changes. The world of work has seen major structural shifts, such as the rise of remote and multilocation work. These developments have increased the flexibility of work and diversified the places in which work is carried out, but at the same time, they have fragmented work communities. The boundaries between work and leisure have become increasingly blurred, and this may lead to higher levels of strain and reduced wellbeing at work.^5,6^ Moreover, the rise in mental health problems in working populations has placed additional demands on OHS, requiring more integrated and responsive approaches.^7^

The shift toward digital health technologies and the delivery of remote services has presented OH professionals with both opportunities and challenges.^8,9^ Although digital tools may enhance accessibility, reduce time and costs, and promote regional equity in the provision of services,^10,11^ they may also complicate communication, reduce face-to-face interactions, and alter traditional care pathways.^12^ These developments require professionals to possess digital competence, the ability to assess clients’ situations remotely, and readiness to integrate technology into interprofessional collaboration.^9,13^

The 2022 legislative reform of the Occupational Health Care Act has influenced the OH operating environment in Finland. The status of OH physiotherapists changed from expert to professional, expanding their opportunities to independently assess clients’ work ability and rehabilitation needs.^14^ For example, a client experiencing musculoskeletal symptoms can now directly access an OH physiotherapist without an OH physician’s referral.^15^

Despite the growing body of research on OH care systems and service delivery models, including studies on the multiprofessional approach^4^ and the use of digital OHS,^16^ there is limited qualitative evidence on how professionals themselves perceive and experience these environmental changes in their daily practice. Understanding these perspectives is essential for developing responsive, sustainable, and client-centered OHS.

OH care systems vary widely between countries, but many face similar pressures related to changes in working life, including digitalization, remote and multilocation work, and increasing demands on mental health. Finland provides a useful case for examining these developments, as its OH care system is legally regulated, covers most of the working population, and combines preventive, curative, and work ability-oriented services.^17^ Although this study was situated in the Finnish context, the findings may be relevant for other OH care systems facing similar challenges.

The aim of this study is to examine OH professionals’ perceptions of the impacts of these changes in the operating environment on OH care practices. By exploring the experiences of OH physicians, nurses, physiotherapists, and psychologists, this study provides timely insights to support the development of customer-oriented services and strengthen multidisciplinary collaboration in OH care.

Our research question is: How do OH professionals perceive the influence of the changes in the operating environment on OH care practices?

## Methods

### Participants and Data Collection

The research data consisted of thematic interviews with 34 professionals, conducted between May and August 2025, as 8 focus group interviews across 8 different OHS providers. We used purposive sampling.^18^ Of the 12 OHS providers we contacted and invited to participate, 8 agreed to take part. The participants were selected via a contact person from each of these pre-selected providers. The selected OHS providers were identified during the recruitment phase and through previous collaboration as organizations operating multiprofessional teams, ensuring that their professionals were able to provide insights relevant to the study aim.

Each focus group was formed within a single OHS provider and consisted of multiple OH professionals. Six focus groups (Groups 1-3, 5-7) represented private providers, 1 was an in-house unit (Group 4), and 1 was a publicly owned company (Group 8). Six groups (Groups 1-3, 5, 7, 8) had an OH physician, an OH nurse, an OH physiotherapist, and an OH psychologist, and 2 groups (Groups 4, 6) differed by having 2 OH nurses. In total, the participants comprised 10 OH nurses, 8 OH physicians, 8 OH physiotherapists, and 8 OH psychologists. Work experience in the current OH team ranged from 1 to 24 years, although 2 participants from Group 7 did not report this information. The participants’ characteristics are presented in Appendix 1.

The interviews covered multiple themes related to multiprofessional preventive OH work, such as the organization of multiprofessional work, documentation practices, information exchange, multiprofessional collaboration, and operating environment changes. This article reports on the findings related to the impacts of the changes in the OH care environment on OH care practices. The questionnaire used in the study consisted of a semi-structured set of interview questions developed specifically for the study. No validated questionnaires were used. The questionnaire was pilot tested with 3 OH professionals who were not among the interviewees to ensure clarity and relevance. The final version of the questionnaire is included as a Supplemental Material. The interviews were conducted via Microsoft Teams, recorded, and transcribed for analysis. The average duration of the interviews was 51 minutes (range 42-57 minutes).

As the analysis focused on responses to a single interview question rather than on developing a comprehensive thematic model, traditional data saturation was not the primary goal. The material was considered sufficient when later focus groups repeated similar perspectives on changes in the operating environment without adding new insights.

Clients were not included, because the focus of the study was on the professionals’ experiences of multiprofessional practices and organizational processes, and this required perspectives specific to OH professionals.

We used Microsoft 365 Copilot Chat to improve the language of the manuscript. The final version of the manuscript was then professionally edited by a native English-speaking language expert prior to resubmission.

### Data Analysis

We identified 194 spoken excerpts from across the focus groups’ responses to the interview question on changes in the OH care environment. From these, we selected 98 excerpts that directly addressed changes in the environment for the analysis and excluded those describing general team practices in order to maintain our focus on the research question.

We used qualitative inductive content analysis to analyze the data, which involved condensing, grouping, and abstracting speech excerpts.^19,20^ First, we read the interview transcripts several times to gain an overall understanding of the data, documenting preliminary analytical observations and contextual reflections as notes.^19^ Second, we identified and coded the meaning units relevant to the study aim, in relation to the research question. Two researchers independently reviewed and discussed the coding to ensure they interpreted it in the same way and that it was consistent, and any differences were resolved through discussion. Third, the identified meaning units were condensed into short descriptive expressions that remained close to the original text. Finally, the condensed meaning units were grouped into subcategories on the basis of content similarity and their alignment with the research questions.^18^ They were then further organized into upper categories representing the main themes.^20^

The results are illustrated by verbatim interview quotations. Although the quotations are labeled by professional background for transparency, the analysis emphasized the professional groups’ shared multidisciplinary experiences rather than their differences. The findings were consistent with the data presented.

The analysis was conducted using Microsoft Excel, which was suitable for organizing the codes, categories, and themes in a dataset of this size and structure. To ensure analytical rigor we used a transparent coding process and researcher collaboration rather than relying on specific software.

## Results

The results are presented in Appendix 2, which shows how we grouped the subcategories from the interview data into 6 thematic categories that reflect the impact of environmental changes on OHS. We now explore each of these 6 themes, focusing on the most significant findings, and supporting them with illustrative quotes from the interviews.

### Stronger Client Orientation in Delivery of OHS

According to the interviewees, the changes in the OH care environment have influenced the planning and delivery of services. Client needs now guide service content to a greater extent than previously. Collaboration with client organizations is improving, and service effectiveness is increasingly monitored using indicators that go beyond cost.

Now we’re developing collaboration with the employer, and the aim is to focus more on impact than euros—what the employer actually gains. Like, if they invest this amount, what do they get? We’re thinking about different indicators together. . . Just to increase the understanding that it’s not all about money. (OH physician, Group_8)

Multidisciplinary work was seen as helping identify client needs, and team strengths were effectively used in client guidance.

Well, I myself have experienced it as really good now, that we’ve more often gone as a multidisciplinary team into these companies. . . And then they’ve become familiar to me too. Then it’s easier for them to express their needs to us. (OH physician, Group_5)

The interviewees reported that preventive OH activities had increased, and that service delivery methods had diversified. Professionals can now influence how services are delivered. The involvement of OH psychologists and physiotherapists in client work has grown, and their role in service planning and delivery has strengthened. Multidisciplinary approaches were considered better, especially when developing care pathways.

. . .for us, care pathways are a concrete step. In preventive individual work, we involve OH psychologists and physiotherapists more. They work in parallel with us and as a clear part of the process in, for example, recovery pathways. (OH physiotherapist, Group_2)

It was noted that the need to inform clients of the full range of services had increased. Awareness of the multidisciplinary team had helped clients contact the right professionals directly. However, more communication about available services is still needed. The interviewees also mentioned plans to develop joint consultations, which would allow clients to meet several professionals during 1 appointment.

. . .our big goal is to hold joint consultations, with doctors, psychologists and, if needed, nurses, for clients with multiple issues. We haven’t had the resources to pilot it yet though, but we haven’t forgotten it. (OH nurse, Group_3)

According to the interviewees, environmental changes had increased the need for individually tailored OHS. Young employees in particular were seen to need support when integrating into working life, which had led to the greater involvement of social work professionals. Similarly, the need for work ability coordinators had grown among older employees.

And maybe one end is young people who haven’t found their place. They might be in a job, but the wrong one, and they haven’t completed their studies yet. Life is complicated. Then it could be a social work professional who could help them make a concrete plan for moving forward. (OH physiotherapist, Group_4)

. . .these changes, especially among older employees, are increasingly creating work for our work ability coordinators. This sector is clearly growing. (OH physiotherapist, Group_2)

The interviewees described client awareness of the services available to them as varying. Even when the OH team composition was presented to the client, some professionals remained unfamiliar to them. Therefore, service content and professional roles were now communicated more actively. When clients knew the team and their expertise, they could seek help from, for example, a physiotherapist or psychologist without a referral. This was seen as speeding up access to appropriate services.

I think this multidisciplinary teamwork starts with us becoming familiar to the company and vice versa. Then it’s easier for them to express their needs. (OH physiotherapist, Group_5)

The interviewees also highlighted how multidisciplinary work has helped them identify client needs and find solutions. Internal team communication and consultation were described as smooth, which enabled quicker responses to client situations.

I guess it’s easier to trace, reflect on, and investigate things when you can share and think them through in a multidisciplinary way. (OH nurse, Group_4)

It emerged that although the need for multidisciplinary participation in workplace surveys was recognized, clients often limited the number of participants due to costs. Especially in smaller workplaces, involving a second OH professional or expert required clear justification.

Yes, I know the nurse almost has to write a poem to get another professional involved, whether it’s a physiotherapist, psychologist or doctor. You really have to justify it with legal clauses and risk assessments, and still the answer is often no. Even if we see it as essential. (OH physiotherapist, Group_8)

Finally, the interviewees said they were now able to decide together with the client whether a consultation was held remotely or in person.

. . .we get to decide ourselves. But we can also discuss with the follow-up visit with the client, whether it could be done remotely. (OH physiotherapist, Group_5)

### Adaptation of OH Care Practices to Multilocation Client Organizations

The interview data indicated that the increasing prevalence of multilocation work had significantly influenced the delivery of OHS. The interviewees noted that workplaces expected services to be equally accessible to all employees, regardless of their location. To meet this expectation, collaboration with other OHS providers had made partnership agreements, to ensure service coverage across different sites.

Many client companies with sites around Finland want consistency, so that the quality of OH care is relevant and equal for all employees and in all locations. (OH physiotherapist, Group_3)

And then these different towns, remote systems are used. And of course, since we now operate in several locations, our clients may have sites across Finland, so we make partnership agreements with another provider, but we remain the coordinating party. (OH nurse, Group_6)

The interviewees described how multilocation client organizations have enabled professionals to work remotely. Remote work has become a routine part of OH care practice and is seen to support both client relationship management and multidisciplinary collaboration, especially with organizations operating in various locations.

I work with large client organizations, and a few of the ones I coordinate are everywhere except here. So there’s a lot of remote work, and that wouldn’t have been possible a few years ago. It’s had a big impact, in a good way. (OH physician, Group_5)

Multilocation work was said to require more careful planning and method selection for workplace surveys. As employees no longer work solely in traditional environments, surveys have been adapted to include remote work settings, and working conditions are thus sometimes assessed at employees’ homes.

At least for me, when it’s multilocation work or unclear where the work is being done, the nurse and I figure out together how to do the workplace surveys. We probably have to plan the methods more carefully than in a traditional workplace, how to collect the data. (OH physician, Group_2)

Yes, for many doing remote work, ergonomics has improved over time, but the beginning was awful. Working at the corner of the kitchen table. We’ve visited home workstations when needed. (OH physiotherapist, Group_6)

### Technology-Driven Development of OH Care Practices

The interviewees reported that the use of digital tools had increased, which in turn has enhanced client work and facilitated collaboration within OH multidisciplinary teams. Microsoft Teams was mentioned as a key tool for quickly arranging meetings and contacting colleagues. Technology was seen as facilitating communication, particularly when team members worked in different locations.

Well at least I think we’re really active on Teams and almost everyone has it open. If you have a question, you can get an answer quickly. And of course we use it to set up meetings, like with the OH psychologist or physiotherapist or nurse, I’ll just join for a moment and ask if we can talk about this. (OH physician, Group_5)

Technology has changed how work is carried out. The interviewees noted that the improved information systems helped identify work ability risks and monitor sickness absence. They also made it easier to recognize clients who need support. Data collection is faster and less manual, as online pre-questionnaires have replaced paper forms.

. . .like data collection, for example, for workplace surveys, with all these electronic pre-questionnaires and tools, we get the data easily and the system calculates it. The information flows better and can be summarized more easily. That’s how I see it has made a difference. There’s not so much paper and pen work anymore. . . you can fill in a survey directly on the computer, it calculates the score, things like that, it’s definitely made things smoother and faster. (OH psychologist, Group_6)

The interviewees also said that technology had changed how clients are supported. Communication between clients and OH professionals now increasingly takes place through digital channels. In some cases, when a client contacts an OH nurse, for example, another OH professional, such as a physician, can be added to the conversation to continue handling the issue.

Well yes, in electronic messaging, when replying, if we see it’s something for the doctor, I just add the doctor to the conversation, and they take it from there. (OH nurse, Group_4)

Variation was noted in clients’ digital skills. While some found digital services easy to use, others struggled. This increased the need for OH professionals to guide and support clients in their use of digital tools.

But yes, there are also clients who find digitalization really difficult. Then we guide them step by step, like how to start the appointment and so on. There’s quite a lot of variation. (OH physician, Group_4)

### Diversified Remote OH Care Practices

The rapid expansion of remote services, especially after the COVID-19 pandemic, has influenced OH operations in many ways. Based on the interviews, remote services have become a standard part of service provision, although their use varies depending on the service content and the client’s situation. Clients may also be skeptical about remote services, but this usually fades after they try them out in practice.

Yes, and there can be a kind of preconceived attitude, but once you see how it works, then it’s actually fine. You come across that. But luckily, there are only a few people who are really skeptical, most are already well on board. (OH nurse, Group_3)

Remote services were seen to mainly focus on clinical care. However, in cases such as musculoskeletal symptoms, the professionals preferred the first appointment to be in person so that they could observe the client’s movement more accurately. Follow up could then be conducted remotely.

. . .that the first appointment is face-to-face, because observation and movement assessment can’t be done via a screen. But then we can discuss with the client what the follow up will be, if it’s something that works remotely. (OH physiotherapist, Group_5)

According to the interviewees, work ability negotiations are now mostly conducted remotely. However, physical presence is still considered important in particularly challenging situations. In such cases, documentation and practical matters are often handled more efficiently.

Almost all work ability negotiations are now remote. If a negotiation is expected to be difficult, then it’s held face-to-face, but otherwise, over 90 percent are remote. Of course, sometimes the patient is here in person and others join via Teams. There’s often paperwork after the negotiation, so it’s easier to handle it right away with the patient. (OH physician, Group_8)

The interviewees noted that remote services had enabled OHS to offer their services regardless of time and place. Accessibility had improved, especially in areas where OHS were previously limited. The increase in remote services was also seen to improve cost-efficiency. Clients could attend appointments or negotiations from their workplaces, reducing the need to travel.

. . .this remote service might also be a more cost-effective option for the client in changing situations—the employee can join from the workplace, so there’s no need to drive back and forth. (OH physiotherapist, Group_3)

The easy accessibility of remote services has also brought challenges. According to the interviewees, it was too easy for some clients to make remote appointments, for example, via chat. This could lead to using services unnecessarily or issues being addressed medically even when a medical assessment was not required.

. . .in some client cases, I think the service is used a bit unnecessarily, because it’s so easy to start a chat or whatever, or a short remote appointment, a call, which is of course really good. . . but sometimes it can lead to over-medicalization, something that could be handled by a familiar OH nurse who understands the work and what’s going on. (OH nurse, Group_1)

The increase of remote work has also changed the working methods of OH professionals. For example, the need for care is increasingly assessed remotely. Multidisciplinary collaboration now often takes place via remote connections, which has made communication between professionals in different locations easier. At the same time, shared in-person work has decreased. One reason identified for this was the limited physical workspace in OH units.

If you knock on the door and get no answer, you might still reach someone via Teams or hold a meeting that way, it’s maybe easier. And you can work not only with teams in your own location, but also with people in other places. Multidisciplinary work with others in different locations has become possible because Teams and other tools work well. (OH psychologist, Group_5)

We have a limited number of rooms available, which partly explains the shift to remote work. Of course, we’d prefer to all be on-site on the same day. (OH physician, Group_7)

The interviews revealed that although using remote services while at home had become more common among clients, OH professionals still mostly conducted remote appointments from consultation rooms. According to the interviewees, the lack of space and equipment needed to demonstrate movements made working from home challenging. Moreover, the number of remote appointments was not always enough to fill a working day.

OH physiotherapists are generally on-site. Occasionally we have an office day or a training day, but not actual physiotherapy work. I said that there are so few remote appointments that they don’t fill a whole day. And then, is everyone able to do quality remote work from home via video and to show movements, have the equipment? So they’re usually in their own consultation rooms. (OH physiotherapist, Group_8)

The interviewees noted that working on-site was often the preferred option, even when remote work was possible. Especially in mental health and substance use cases, they considered it more difficult to understand the client’s overall situation remotely than in face-to-face meetings.

Those remote appointments are both good and bad. In substance use cases, very bad. . . For identifying issues, they’re absolutely useless. (OH physician, Group_1)

### Stronger Mental Health Support Structures

According to the interviewees, the need for the expertise of OH psychologists is growing, and the demand for their services has increased. This has led to both congestion in psychologists’ consultation schedules and an increase in the number of professionals. The use of remote services in psychologists’ consultation work has grown.

Well, yes, we increasingly have to rely on our OH psychologists, and their diaries are starting to look quite full. (OH nurse, Group_6)

Access to mental health services has improved, as clients now encounter fewer barriers to seeking help from OH psychologists. The interviewees also noted that workplaces now contact psychologists more actively than before. Furthermore, psychologists are increasingly involved in organizational-level work alongside individual consultations, such as workplace surveys and work community support.

From the psychologist’s perspective, you can definitely see that organizations are reaching out more and asking for advice or lectures on, for example, how to talk about difficult topics related to mental health problems or sickness absences that might be due to mental health issues. (OH psychologist, Group_5)

The interviewees reported that multidisciplinary team collaboration on mental health matters had increased. Team meetings now more frequently address themes related to psychological workload. Collaboration among OH psychologists themselves was also perceived to have strengthened, especially when similar symptoms were observed among several employees in the same workplace. In such cases, the psychologists aimed to jointly identify the underlying factors and determine what kind of services could be offered in response.

These changes have brought new themes into our team collaboration. . . (OH physiotherapist, Group_1)

We do try to look into it together as psychologists, especially if a particular workplace is sending a lot of clients. Then we react and ask what’s going on there. Or if there are similar kinds of symptoms. (OH psychologist, Group_8)

The interviews revealed that work ability negotiations related to mental health were now initiated earlier than before. However, they emphasized that more use should be made of the expertise of OH psychologists to support supervisors.

If something more is needed, then what could that be? And sometimes we’ve held work ability negotiations together, or lighter versions of them in situations directly related to workplace matters—and in these cases, I think it’s worked really well. (OH physician, Group_7)

. . . Somehow there should be some kind of psychological support or something that could be offered to the supervisor at that point. . . (OH physician, Group_5)

### Changed Role of OH Physiotherapist in Delivering Services

According to the interviewees, the transformation of the OH physiotherapist’s role from an OH expert to an OH professional has enabled clients to access a physiotherapist directly without a referral from an OH physician or nurse. This has improved the availability of services and reduced unnecessary intermediaries. However, the interviewees highlighted that client communication needed strengthening, as some clients were still unaware that they could consult an OH physiotherapist directly. In addition to informing clients of this, internal team communication also needs to be enhanced.

It’s taken time for this to become embedded among clients, but they’re increasingly aware of it. . . but you could still basically mention it every day, that this direct option now exists. (OH physiotherapist, Group_1)

But it means we have to talk to each other. Keep the team informed. (OH physician, Group_1)

The interviews revealed that in some OH units, clients with musculoskeletal symptoms were now primarily directed to the OH physiotherapist. As a result, clients now mainly consult the OH physician when their symptoms become prolonged.

. . . musculoskeletal patients hardly ever come to the physician before they’ve seen the OH physiotherapist. Then it’s the prolonged cases. If a back patient comes to the physician for an initial visit, that’s rare. (OH physician, Group_8)

The interviewees reported that this role change has increased OH physiotherapists’ responsibilities in managing client relationships and in organizational work. Physiotherapists now support workplace work ability management more actively by, for example, monitoring sickness absences. They can also act as primary participants in workplace surveys, attending on-site if the team so decides, while other professionals participate remotely.

But now, being a healthcare professional, we do work ability management and monitor sickness absences and carry out our part and manage cases just like the OH nurse. (OH physiotherapist, Group_3)

. . . physiotherapists can now also carry out workplace surveys. We can decide on a case by case basis who it makes the most sense to send on-site, and who joins remotely via video, for example. (OH nurse, Group_3)

However, it seems that this change in roles had not taken place uniformly across all the interviewed OH teams. Despite joint planning, in some cases, the number of joint basic workplace surveys had decreased. This was partly seen as being because workplaces limited participants for cost-related reasons. The OH physiotherapist only attended on-site surveys in particularly demanding cases. The interviews also showed that referring clients to an OH psychologist had not yet become a routine part of the physiotherapist’s work, even though it was now possible.

. . . that OH physiotherapists are quite easily excluded. We rarely get to participate in basic workplace surveys, even when risk assessment shows it would be justified. Then they tend to be focused on workplace surveys, carried out independently, not together with the rest of the team. But that doesn’t stop our joint planning here. (OH physiotherapist, Group_8)

There hasn’t yet been a single referral to an OH psychologist from a physiotherapist. (OH psychologist, Group_7)

According to the interviewees, the role transformation has increased OH physiotherapists’ decision-making power regarding service delivery. They felt that physiotherapists now had more opportunities to influence how services are organized. This was reflected in, for example, participation in joint team planning or being able to agree directly with the client whether the service is delivered in person or remotely. The change was seen as bringing more flexibility to the physiotherapist’s work. However, the interviewees also emphasized a need to strengthen client trust in the physiotherapist’s role. It was considered especially important that clients feel that the physiotherapist is capable of handling their issue independently and will involve a physician when necessary.

We don’t have strict definitions for how the work is done—we can decide ourselves whether the appointment is in person or remote. (OH physiotherapist, Group_5)

We have to convince the client that the OH physiotherapist will involve a physician when needed—trust needs to be built. (OH physiotherapist, Group_1)

## Discussion

This study examined OH professionals’ perceptions of the impacts of changes in the operating environment on OH care practices.

Our findings indicate that remote services have become an established part of OH care. They have improved service accessibility and cost-efficiency. These results support the findings of previous studies that remote services enhance access to care regardless of location and may reduce both service and travel costs.^9-11,21-23^ However, as the interviewees reported, face-to-face appointments are still necessary in situations where movements need to be observed, in mentally demanding and substance use-related cases, and in challenging work ability negotiations, because understanding the client’s overall situation is more reliable in person.

Our findings also reveal that some clients may feel uncertain about remote services. However, smooth user experiences^24^ and perceived benefits^25^ increase both satisfaction and usage. Professionals often express concern that clients’ limited digital skills can hinder the use of services.^9^ Successful use of remote services relies on support^26,27^ and encouragement from professionals.^25^ This study confirms that clients’ digital skills are often insufficient and require guidance and encouragement from professionals. The active effort to support clients reflects the growing emphasis on client-centeredness in the evolving OH care environment. However, remote consultations also require adequate skills from OHS professionals, who must be able to evaluate whether a client’s situation is suitable for remote care and ensure that the chosen mode of delivery supports both safety and clinical quality.

According to the findings, collaboration within multidisciplinary teams is increasingly taking place via remote connections, and this has facilitated information exchange between professionals working in different locations. One reason attributed to this reduction in shared physical presence was the lack of workspaces. In such cases, remote work appears to be used out of necessity, which may not fully align with the employer’s obligation to provide appropriate work facilities.^28^ At the same time, as the shift to remote connections may weaken professionals’ sense of community and reduce social support from colleagues, it is important to pay attention to how interaction and the prerequisites for teamwork are maintained in this new dispersed work environment,^29^ and to ensure that remote work remains voluntary for professionals.

Salmela et al^30^ found that early support is a key factor in promoting the work ability of employees who experience psychological strain and in preventing sickness absences. This highlights the important role of OHS in the early identification of mental health problems and in providing support. In addition to individual-level support, workplaces should be made aware of the work community’s mental health and well-being, and of workplace-level measures.^31^ In our study, the participants described an increased emphasis on mental health services within OH care, particularly through the greater utilization of OH psychologists. Utilizing OH psychologists was seen as a central part of supporting work ability, and the number of psychologists has increased, which supports the core goals of OHS related to early support and the promotion of employees’ work ability. In addition, as Lahti et al^32^ have shown, individual support from OH psychologists can significantly reduce mental health-related sickness absences. Notably, OH psychologists are the only professionals in the multidisciplinary OH team whose services continue to require a referral.

Our findings indicate that OH psychologists’ organizational-level work has increased. They are increasingly involved in workplace surveys. This development is likely to have been influenced by the 2023 clarification of employers’ obligations to assess psychosocial factors at work, such as job content, work arrangements, and the social functioning of the work community.^33^ These developments reflect the Finnish OH care system. International comparisons show that Finland has more extensive, legally defined employer responsibilities, whereas in many other countries (eg, the UK, the US, Australia), OH care obligations are less comprehensive and less regulated.^17^ OH psychologists also more actively support work communities. However, research evidence on effective workplace-level interventions remains limited,^34^ and thus further research is needed.

The shift in the professional status of OH physiotherapists to recognized healthcare professionals has increased their responsibilities and authority to make decisions about the delivery of services, and enabled more flexible service organization. This change has expanded their responsibilities in client relationship management and organizational work. At the same time, the need to strengthen clients’ trust in the independent role of the OH physiotherapist has become more pronounced. The change has also increased OH physiotherapists’ involvement in work ability management and enabled clients to contact them directly. Being able to access OH physiotherapist services directly has accelerated clients’ access to care, and unnecessary use of these services has not increased.^15^

International comparisons indicate that the role of physiotherapists in OH care varies between countries.^17^ In more regulated systems such as that in Finland, physiotherapists are integrated into multidisciplinary OH teams and are involved in workplace surveys and work ability management, whereas in less regulated systems their role is more limited and focuses on clinical treatment. Although the findings of our study mainly reflect the Finnish context, they may inform countries seeking to strengthen the integration of physiotherapy into OHS.

Based on our findings, the changes in the professional status of OH physiotherapists have intensified information exchange within OH teams. However, the nature of teamwork appears to align more closely with a multidisciplinary approach rather than a deeper interprofessional model. In a multidisciplinary team, professionals work independently and report their findings to the physician, whereas in an interprofessional team, each professional conducts discipline-specific assessments and then collaborates to discuss and coordinate the care plan.^35^ However, some professionals perceived that the change in professional status had not affected OH care practices or had even led to a decrease in multidisciplinary collaboration. This suggests a need to foster trust within the OH team, although further investigation is required to confirm this interpretation.

The findings of this study suggest that strengthened multidisciplinary collaboration, the expanded roles of OH psychologists and OH physiotherapists, and the increased use of remote services can improve timely access to support and enhance early identification of work ability risks. At the same time, the results highlight situations where in-person assessment remains essential, underscoring the need for careful choice of service delivery mode in OHS.

### Strengths and Limitations

Inductive content analysis was chosen as the method to explore the phenomenon openly and from the participants’ perspectives, without predefined theoretical constraints. This approach was particularly appropriate given the limited prior research and theoretical modeling on the topic.

The quality of content analysis depends largely on the coding process.^36^ In this study, the first author coded the data in accordance with the study aim, grouping meaning units by content similarity. The second author reviewed all the units and their categorization to enhance validity, and the third author contributed through discussions on the findings.

In inductive content analysis, researchers’ professional backgrounds and theoretical orientations influence how data is interpreted.^19^ While the researchers’ strong expertise in OH supported the identification of relevant phenomena and contextual understanding, it may also have shaped the emphasis and interpretation of the data. To mitigate this, reflexivity was emphasized throughout the analysis to ensure that the interpretations remained grounded in the data rather than on preconceptions.

Another limitation relates to the variation in the participants’ work experience, as some had relatively little experience in OH care. However, their views were complemented by those of more experienced colleagues in the group interviews.

In addition, because participants were recruited through a designated contact within each OHS provider, the selection process may have favored professionals who were more readily available or willing to participate, which may introduce a minor selection bias.

A further methodological limitation is that the questionnaire consisted of a study-specific set of semi-structured interview questions rather than a previously validated instrument. Although the questionnaire was pilot-tested with 3 OH professionals to ensure clarity and relevance, its study-specific nature may limit direct comparability with other qualitative studies.

The exclusion of clients’ perspectives may also be considered a limitation, and future studies could examine clients’ experiences of changes in OH care practices.

## Conclusion

This study shows that OH professionals in Finland are actively adapting to structural changes in their operating environment. Remote services have become a routine part of OH care, and although they improve accessibility and cost-efficiency, they also highlight a need to support clients with limited digital skills. Multidisciplinary collaboration has increasingly shifted to virtual platforms, enabling broader participation across locations. However, this transition may challenge team cohesion and shared practices, and attention should be paid to maintaining interaction and continuity in teamwork. The growing demand for mental health services has expanded the role of OH psychologists, who now contribute more actively to both individual care and organizational-level interventions. Similarly, the redefinition of OH physiotherapists as independent healthcare professionals has enhanced the flexibility of services, accelerated access to care, and expanded OH physiotherapists’ role in work ability management and organizational collaboration. Strengthening client trust remains essential, but evolving roles may also require building trust within the OH team. This warrants further research into the development of collaborative practices from the perspective of interprofessional teamwork. The results suggest that OH professionals are responding to environmental changes by developing more client-centered, flexible, and collaborative service models.

## Supplemental Material

sj-docx-1-his-10.1177_11786329261447088 – Supplemental material for Perceptions of Changes in the Operating Environment and Their Impact on Occupational Health Care Practices: A Qualitative Interview Study Supplemental material, sj-docx-1-his-10.1177_11786329261447088 for Perceptions of Changes in the Operating Environment and Their Impact on Occupational Health Care Practices: A Qualitative Interview Study by Sari Nissinen, Mari-Anne Wallius and Erja Sormunen in Health Services Insights

sj-docx-2-his-10.1177_11786329261447088 – Supplemental material for Perceptions of Changes in the Operating Environment and Their Impact on Occupational Health Care Practices: A Qualitative Interview Study Supplemental material, sj-docx-2-his-10.1177_11786329261447088 for Perceptions of Changes in the Operating Environment and Their Impact on Occupational Health Care Practices: A Qualitative Interview Study by Sari Nissinen, Mari-Anne Wallius and Erja Sormunen in Health Services Insights

## Appendix

**Appendix 1.** Characteristics of Focus Group Participants.

Focus group	Type of OH service provision	Participant	Work experience in current OH team (y)
1	Private OH service provider	OH psychologist	4.5
		OH physiotherapist	25
		OH physician	6
		OH nurse	5
2	Private OH service provider	OH nurse	6.5
		OH physiotherapist	10
		OH psychologist	5
		OH physician	11
3	Private OH service provider	OH physician	10
		OH nurse	8
		OH physiotherapist	20
		OH psychologist	5
4	In-house OH provider within a corporate	OH nurse	29
		OH nurse	18
		OH physician	17
		OH physiotherapist	9
		OH psychologist	4
5	Private OH service provider	OH psychologist	4
		OH physiotherapist	19
		OH physician	13
		OH nurse	20
6	Private OH service provider	OH nurse	30
		OH physician	32
		OH psychologist	4
		OH physiotherapist	35
		OH nurse	36
7	Private OH service provider	OH physician	13
		OH psychologist	Not reported
		OH physiotherapist	Not reported
		OH nurse	13
8	Publicly owned company	OH physician	18
		OH psychologist	6
		OH nurse	13
		OH physiotherapist	21

**Appendix 2.** Subcategories From Interview Data Grouped into 6 Thematic Categories Describing the Impact of Environmental Changes on Occupational Health Care Practices.

Subcategory	Thematic category
Client needs have expanded service delivery	Stronger client orientation in delivery of occupational health services
Multidisciplinary work has made it easier to identify client needs	
Clients have limited the use of multidisciplinary work	
Multidisciplinary services are now better utilized in client guidance	
Recognized need to develop joint professional consultations for clients	
Increased need to strengthen preventive work through multidisciplinary collaboration	
Increased need to inform clients of the range of available services	
Effectiveness monitoring has become part of client collaboration	
Multidisciplinary perspectives are better integrated into care pathway development	
Familiarity with multidisciplinary team has increased direct client contacts	
Professionals have greater influence over way in which services are delivered	
Clients’ multilocation work has increased the need to plan workplace surveys	Adaptation of occupational health care practices to multilocation client organizations
Clients’ multilocation work has increased the use of remote services	
Client’s multilocation work has enabled professionals to work remotely	
Workplace surveys have expanded to include remote work environments	
Technology has facilitated smoother multidisciplinary collaboration	Technology-driven development of occupational health care practices
Impact of technology use on work practices	
Impact of technology on client relationship management	
Use of technology in services requires professionals to guide clients	
Impact of remote work on multidisciplinary collaboration	Diversified remote occupational health care practices
Impact of COVID-19 pandemic on increase in remote services	
Remote services have improved service accessibility	
Impact of remote services on the way in which services are delivered	
Impact of remote services on client collaboration	
Impact of work tasks on implementation of remote services	
Impact of limited physical workspace on work arrangements	
Impact of remote services on service usage	
Impact of remote services on service quality	
Impact of remote services on multidisciplinary work	
Impact of remote services on identifying mental health symptoms	
Positive impact of remote services on client costs	
Clients directed to remote services on incorrect grounds	
Remote services have been limited to clinical care	
Client attitude more positive after using remote services	
Impact of number of remote clients on work arrangements	
Increased need for occupational health psychologist services	Stronger mental health support structures
Increase in number of occupational health psychologists	
Increased adoption of remote services in occupational health psychologists’ work	
Easier access to mental health services	
Earlier initiation of mental health-related work ability negotiations	
Requirement to assess psychosocial risks has increased psychologists’ organizational-level work	
Mental health services have become more multidisciplinary in response to challenges	
Occupational health psychologists’ increased participation in workplace surveys	
Lower threshold to seeking help from occupational health psychologist	
More frequent team discussions on mental health problems	
Change in occupational health physiotherapist’s professional role has affected multidisciplinary work	Changed role of occupational health physiotherapist in delivering services
Increased need to raise awareness of occupational health physiotherapy services among clients	
More initial appointments for musculoskeletal patients with occupational health physiotherapist	
Multidisciplinary collaboration has decreased following change in professional status	
Greater availability of occupational health physiotherapy services	
Changes in professional status of occupational health physiotherapists have intensified information exchange in multidisciplinary collaboration	
Occupational health physiotherapist direct more workplace surveys	
Change in occupational health physiotherapist’s professional role requires building client trust	
Change in professional status has improved client-driven service use	
Change in professional status has improved client-centered practice	
Occupational health physiotherapists more involved in managing client relationships	
