# Supplementary material for: Perceptions of Changes in the Operating Environment and Their Impact on Occupational Health Care Practices: A Qualitative Interview Study
Source: Health Serv Insights. 2026 May 5;19:11786329261447088. doi: 10.1177/11786329261447088 (PMC13161666; doi:10.1177/11786329261447088)
Supplement: sj-docx-2-his-10.1177_11786329261447088 – Supplemental material for Perceptions of Changes in the Operating Environment and Their Impact on Occupational Health Care Practices: A Qualitative Interview Study [file sj-docx-2-his-10.1177_11786329261447088.docx]

**Supplementary**

**Semistructured Interview Questionnaire (Translation from Finnish)**

1. What is your first name, professional title, and work experience in your current occupational health unit as well as your overall work experience in occupational health care?
2. Do you give your consent to participate in the study and to have the interview recorded, with the data used for reporting purposes?
3. Please describe the multiprofessional practices in preventive occupational health work in your occupational health unit (e.g., levels and forms of collaboration)
   1. How have multidisciplinary collaboration and professional roles been agreed upon within your unit? Who is responsible for coordinating the collaboration?
   2. How systematic is your multidisciplinary practice? Is dedicated time reserved for multidisciplinary collaboration and for planning that collaboration?
   3. Are there differences between large and small client workplaces?
4. How have changes in working life influenced multiprofessional practices in occupational health care?
   1. In what ways have multilocation work, digitalization, remote/hybrid work, and increasing mental health demands affected the multidisciplinary collaboration within your team?
5. How have changes in the occupational health care operating environment influenced multiprofessional practices? (including the change in the professional status of occupational health physiotherapists and the increased availability and use of remote services)
   1. In what ways have the change in the professional status of occupational health physiotherapists and the increased availability and use of remote services affected the multidisciplinary collaboration within your team?
6. What practices do you have in place regarding the documentation of client data?
7. How is the transfer of client information between different professional groups ensured when the entire multiprofessional team has not been involved?
8. In what ways do your information systems support information exchange and the use of information?
9. What aspects of multiprofessional collaboration work well?
10. What development needs have you identified in multiprofessional collaboration?
11. How could you collectively develop multiprofessional collaboration to better meet the needs of client organizations?
12. What factors promote or support multiprofessional collaboration?
13. What factors weaken or hinder collaboration?
14. What kind of support or training do you feel you need to carry out multiprofessional collaboration effectively?
